# Supplementary material for: Using the Conditional Process Analysis Model to Characterize the Evolution of Carbon Structure in Taxodium ascendens Biochar with Varied Pyrolysis Temperature and Holding Time
Source: Plants (Basel). 2024 Feb 5;13(3):460. doi: 10.3390/plants13030460 (PMC10857065; doi:10.3390/plants13030460)
Supplement: Supplementary file 1 [file plants-13-00460-s001.zip › plants-2811325-supplementary.pdf]

1. ANOVA analysis

Table S1. ANOVA analysis for the effects of feedstock types, pyrolysis temperature, and holding time on the fixed carbon, ash, C, H, O, and N contents in biochar samples.

Predicting variables	Outcome variables					
	**FC**	**Ash**	**N**	**C**	**H**	**O**
	sig.	sig.	sig.	sig.	sig.	sig.
part	0.000	0.000	0.000	0.000	0.191	0.000
tempt	0.000	0.000	0.000	0.000	0.000	0.000
time	0.000	0.000	0.003	0.742	0.001	0.164
part * tempt	0.000	0.000	0.000	0.000	0.151	0.000
part * time	0.000	0.000	0.004	0.314	0.482	0.928
tempt * time	0.000	0.000	0.000	0.001	0.010	0.002
part * tempt * time	0.000	0.000	0.000	0.000	0.170	0.003

Tempt represents temperature; FC means fixed carbon; sig means significance level; $P < 0.05$, ANOVA, Bonferroni's test, n=3.

2. Raman analysis for Evolution in carbon structures of BC and LC

Table S2. Evolution in carbon structures of BC and LC with temperature and time by Raman analysis.

Time	Tempt	Typical carbon structures for the 9 Gaussian bands in Raman spectra (Percentage of band area, %)								
		**D**	**G**	**D_R**	**G_L**	**D_L**	**G_R**	**S**	**S_L**	**R**
Branch-based biochar (BC)										
0.5h	350℃	11.770	31.917	28.144	15.860	11.532	1.670	1.247	0.211	1.052
	450℃	11.652	28.514	25.317	14.075	13.423	1.407	3.303	0.493	0.079
	650℃	20.874	26.516	14.225	11.856	18.373	3.292	5.664	0.474	0.769
	750 ℃	27.432	24.927	9.378	11.401	16.735	1.404	4.016	0.746	0.250
1h	350℃	12.238	28.208	27.288	9.001	9.347	3.641	4.467	2.813	1.237
	450℃	14.925	26.408	23.843	8.929	11.881	3.721	8.734	1.474	2.684
	650℃	28.067	26.628	11.718	8.737	13.326	2.667	9.168	0.745	1.032
	750 ℃	27.054	24.173	9.799	5.356	17.829	2.258	8.949	0.703	0.952
2h	350℃	11.945	27.875	22.879	10.297	8.319	2.655	5.075	1.779	1.144
	450℃	17.441	26.449	20.204	10.201	10.574	2.564	5.208	0.984	1.381
	650℃	24.529	25.628	9.679	7.394	15.867	2.980	7.199	0.610	0.901
	750 ℃	28.076	25.345	8.714	3.882	11.860	1.831	6.126	0.457	0.601
Leaf-based biochar (LC)										
0.5h	350℃	17.668	26.017	24.803	13.751	3.113	4.576	6.464	1.813	0.765
	450℃	19.684	25.017	21.771	13.282	7.083	2.925	7.659	1.183	2.101
	650℃	35.304	23.084	11.797	10.591	11.973	2.621	8.786	0.902	0.674
	750℃	37.684	22.452	7.546	9.962	9.539	1.695	8.307	0.518	0.225
1h	350℃	16.862	27.794	24.803	9.156	7.338	2.807	5.078	2.132	1.119
	450℃	19.956	25.304	20.092	10.790	9.397	3.149	7.388	1.728	2.217
	650℃	34.170	23.057	11.133	12.081	11.165	2.528	9.413	1.022	1.472
	750℃	38.566	23.747	6.898	11.765	9.867	1.590	8.536	0.752	1.038
2h	350℃	14.039	30.572	24.804	10.989	6.822	1.037	5.075	3.081	1.474
	450℃	22.243	27.524	18.413	10.411	11.562	3.374	5.208	1.643	2.334
	650℃	33.036	23.662	10.469	10.248	12.791	2.435	7.199	1.527	2.270
	750 ℃	39.448	21.477	6.250	8.350	12.650	1.484	6.126	0.602	1.851

Tempt represents temperature. Considering the three points on each sample for scanning, the number describing the area percentage of every Raman band was the mean value.

3. Plot for moderating effects on aromatic structures

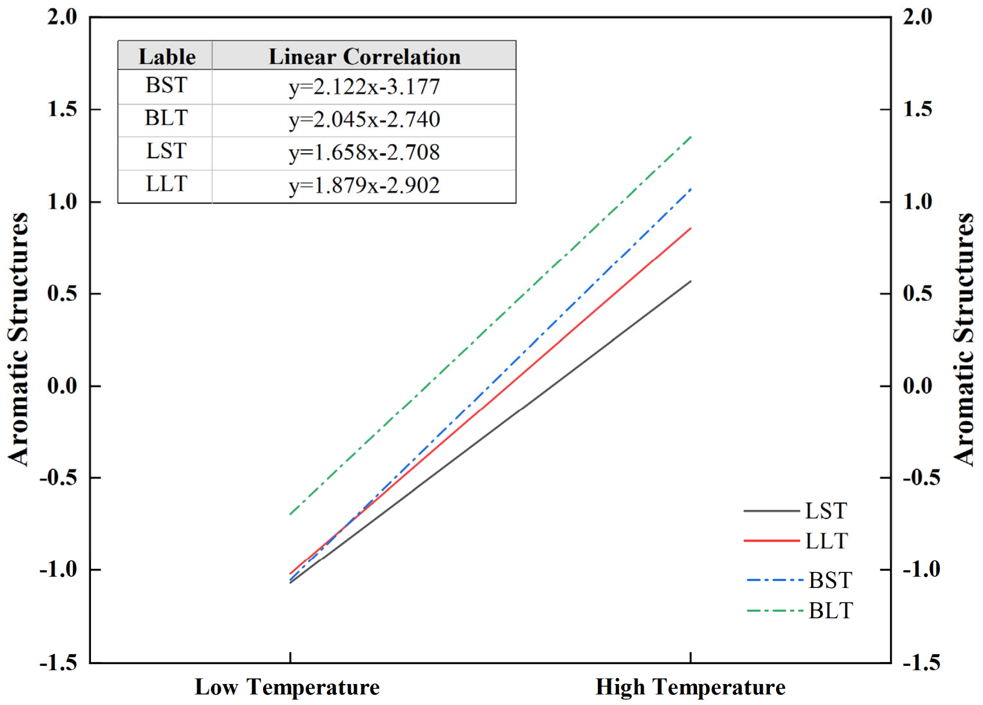

Figure S1. Plot for moderating effects of holding time on aromatic structures of BC and LC: there is a visual representation of the moderation effect of holding time on the association between the aromatic structure and temperature in BC and LC plotted at a standard deviation below the mean (short time = 0.539 hours; low temperature = 390 °C), and a standard deviation above the mean of pyrolysis temperature and time (long time = 1.795 hours; high temperature = 709 °C); BST represents the short hours-pyrolyzed BC; BLT represents the long hours-pyrolyzed BC; LST represents the short hours-pyrolyzed LC; LLT indicates the long hours-pyrolyzed LC.

4. Plot for moderating effects on nonaromatic structures

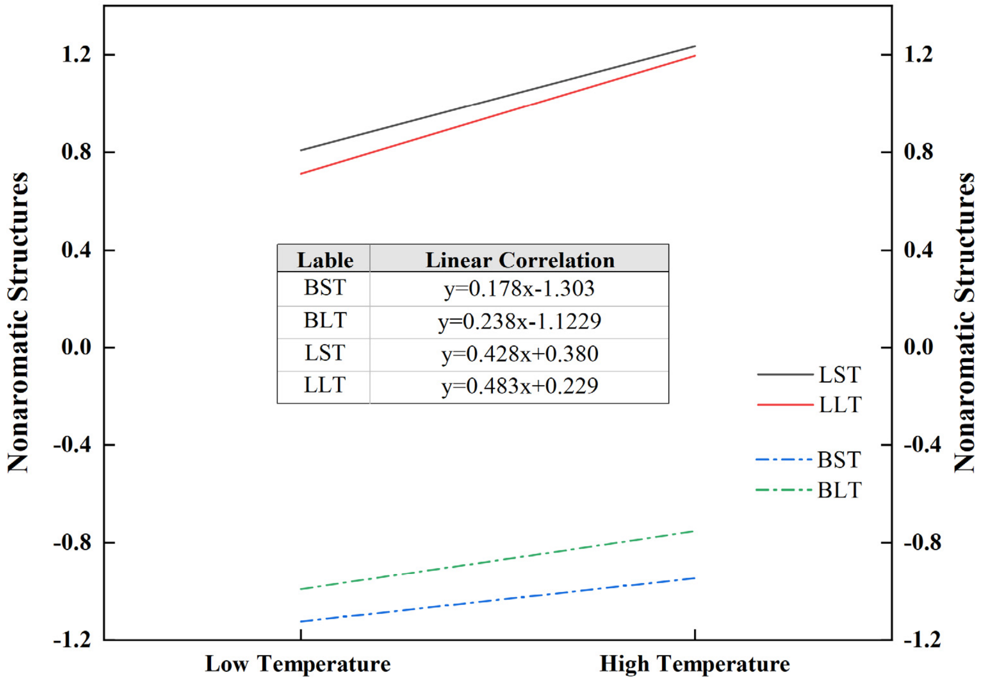

Figure S2. Plot for moderating effects of the holding time on the nonaromatic structures of BC and LC: there is a visual representation of the moderation effect of holding time on the association between the nonaromatic structure and temperature in BC and LC plotted at a standard deviation below the mean (short time = 0.539 hours; low temperature = 390 °C), and a standard deviation above the mean of pyrolysis temperature and time (long time = 1.795 hours; high temperature = 709 °C): BST represents the short hours-pyrolyzed BC; BLT represents the long hours-pyrolyzed BC; LST represents the short hours-pyrolyzed LC; LLT indicates the long hours-pyrolyzed LC.

5. SEM analysis for BC samples

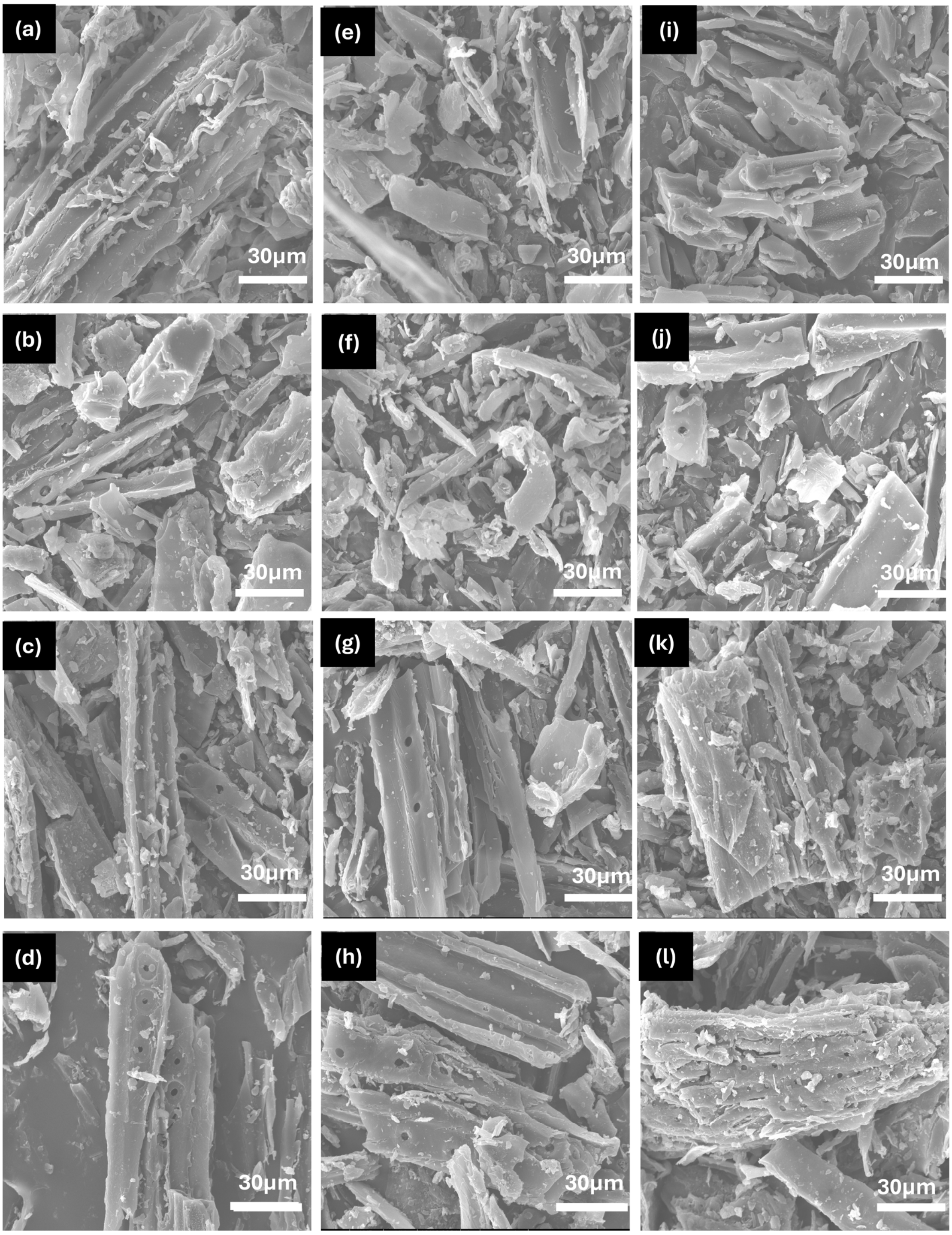

Figure S3. SEM analysis for BC samples: there are SEM micrograph of BC samples at 350 ℃ (a), 450 ℃(b),650 ℃(c), 750 ℃(d) with holding time of 0.5 h, at 350 ℃ (e), 450 ℃(f),650 ℃(g), 750 ℃(h) with holding time of 1 h and at 350 ℃ (i), 450 ℃(j),650 ℃(k), 750 ℃(l) with holding time of 2 h.

6. SEM analysis for LC samples

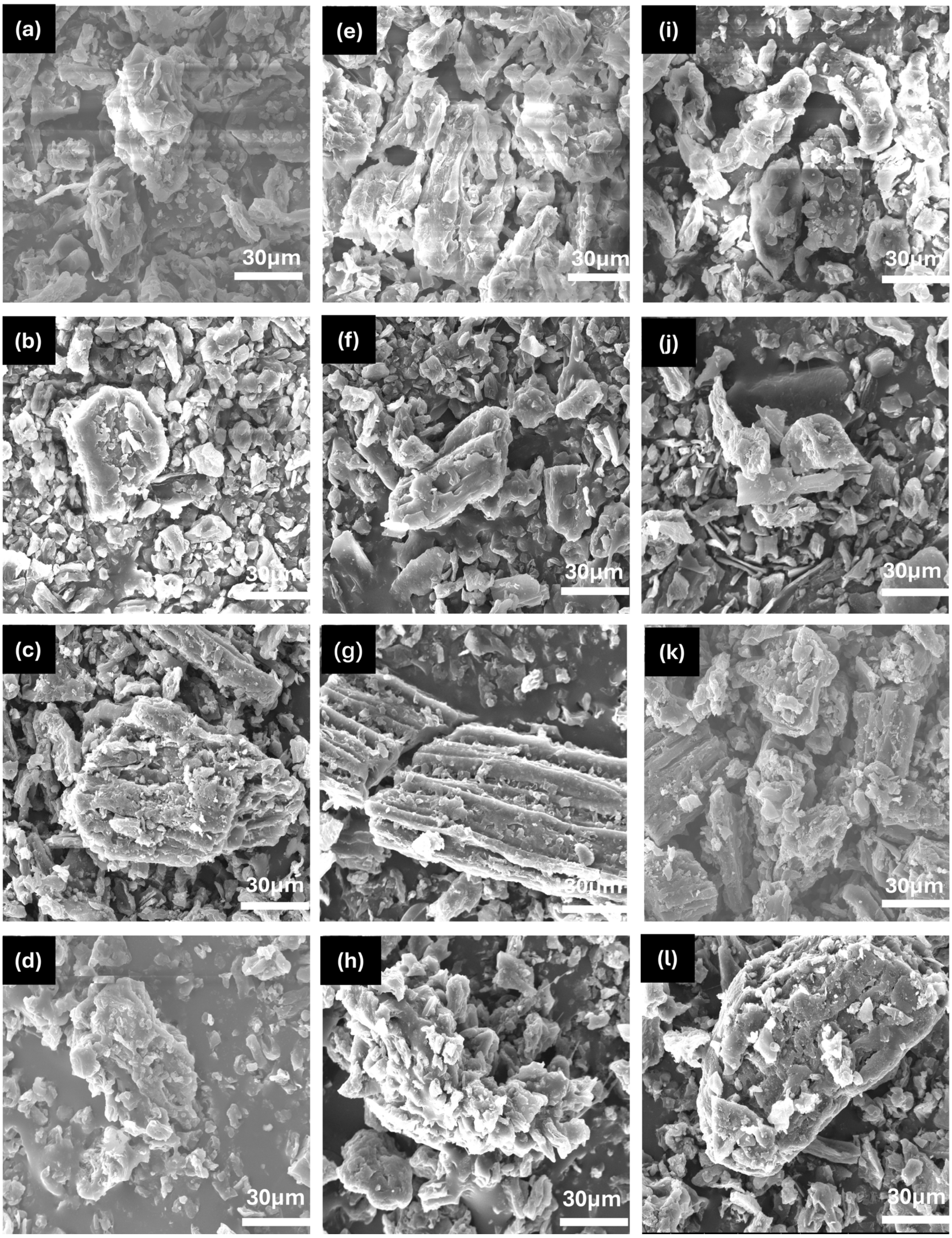

Figure S4. SEM analysis for LC samples: there are SEM micrograph of LC samples at 350 ℃ (a), 450 ℃(b),650 ℃(c), 750 ℃(d) with holding time of 0.5 h, at 350 ℃ (e), 450 ℃(f),650 ℃(g), 750 ℃(h) with holding time of 1 h and at 350 ℃ (i), 450 ℃(j),650 ℃(k), 750 ℃(l) with holding time of 2 h.
